# Exploring the Potential of an Industry-Scale Microfluidizer for Modifying Rice Starch: Multi-Layer Structures and Physicochemical Properties

**DOI:** 10.3390/foods14122067

**Published:** 2025-06-11

**Authors:** Xiaohong He, Zhimeng Yang, Xufeng Wang, Zhou Xu, Yunhui Cheng, Wei Liu, Chengmei Liu, Jun Chen

**Affiliations:** 1 School of Food Science and Bioengineering, Changsha University of Science & Technology, Changsha 410114, China; hexiaohongmaimai@163.com (X.H.); zhimengyang20@163.com (Z.Y.); xufengw@csust.edu.cn (X.W.); xz_jnu@126.com (Z.X.); cyh@csust.edu.cn (Y.C.); 2 College of Food Science, South China Agricultural University, Guangzhou 510642, China; 3 South Subtropical Crop Research Institute, China Academy of Tropical Agricultural Sciences, Zhanjiang 524091, China; 4 State Key Laboratory of Food Science and Resources, Nanchang University, Nanchang 330047, China; liuwei@ncu.edu.cn (W.L.); liuchengmei@ncu.edu.cn (C.L.)

**Keywords:** rice starch, modification, industry-scale microfluidizer, structure, physico-chemical properties

## Abstract

The modification effects of industry-scale microfluidizer (ISM) technology on small-sized rice starch remain unknown. This study systematically evaluated the effects of ISM treatment on the structural characteristics (granular morphology, crystallinity, and short-range order) and physicochemical properties (thermal, pasting, and rheological properties) of rice starch. Scanning electron microscopy (SEM) analysis revealed that ISM treatment induced the aggregation of starch granules, leading to an increase in particle size. Furthermore, ISM treatment resulted in starch damage, as evidenced by an increase in the damaged starch content from 4.25% to 17.99%. X-ray diffraction (XRD) analysis found that the relative crystallinity decreased from 29.01% to 20.74%, and Fourier-transform infrared (FTIR) spectroscopy implied that the absorbance ratio of 1047 cm^−1^/1022 cm^−1^ decreased from 0.88 to 0.73, indicating the disorganization of long-range crystalline structure and short-range ordered structure. Differential scanning calorimetry analysis demonstrated that ISM treatment reduced the gelatinization enthalpy of rice starch, with a gelatinization degree reaching 31.39%. Rapid visco analyzer (RVA) measurements indicated that ISM treatment increased the pasting viscosity of rice starch. However, the effect of ISM treatment on the dynamic rheological properties was minimal, with a slight enhancement in the loss modulus, while in-shear structural recovery rheology showed no significant impact on the ability of starch gels to recover their original structure. These results suggested that ISM technology effectively modified rice starch, leading to a disrupted structure, increased viscosity, and preserved gel network structure. This approach offers a novel strategy for the application of industry-scale microfluidizers in the development of rice-based products.

## 1. Introduction

Starch is a distinctive copolymer characterized by its unique physicochemical properties. Its low cost, biodegradability, renewability, and widespread availability make it an invaluable resource in the food industry [1]. However, native starch exhibits several limitations, including poor solubility, insufficient functionality, and instability under varying temperature and pH conditions, which often render it unsuitable for specific industrial applications [2]. Consequently, starch modification is essential to enhance its processing performance, and the development of effective, feasible, and environmentally friendly physical modification techniques aligns with the growing consumer demand for sustainable, safe, and clean-label products [3,4]. Some green technologies, such as ultrasound, microwave, and cold plasma, have been widely reported for modifying starch. For example, the structure and physicochemical properties of buckwheat starch were modified by ultrasound using different ultrasonic powers combined with moisture contents [5]. Sun et al. modified rice starch via microwave and cold plasma treatments [6]. However, most of these techniques are not continuous and high-volume processing industrial-scale equipment.

Industry-scale microfluidizer (ISM) is an emerging continuous physical processing technology. Previous studies have provided comprehensive diagrams and explanations of its operational principles [7]. The equipment is distinguished by large-diameter microchannels, high processing capacity, and distinctive impact modes that generate complex mechanical effects [8,9]. These features address the limitations of traditional dynamic high-pressure microfluidizer reactors, which are constrained by small chamber sizes and limited processing capacity [10]. Building on the ability of industry-scale microfluidizers to generate high-density energy through large-diameter microchannels, our previous research employed ISM to modify large-sized potato starch and medium-sized pea starch. The results demonstrated that ISM could fragment large potato starch granules and produce partially gelatinized pea starch granules while also significantly modulating their pasting and rheological properties [7,11]. However, the modification effects on smaller starch granules remain unkown. Rice starch is main component of rice, which is the most important cereal to serves as a staple food for more than half of the world’s population [12]. It is a representative small-sized starch with hypoallergenic properties, neutral taste, and bright white color, which makes it widely utilized in developing rice-based foods [13]. Meanwhile, its characteristic of fine particle size that is comparable to fat globules could effectively mimic the mouthfeel of fat, making it particularly valuable as a fat replacer in various food products [14]. Nevertheless, native rice starch still faces several limitations such as high sensitivity to shear forces and poor water solubility, which restrict its widespread application in the food industry [15]. As a time-saving industrial-scale green processing technology, the ability of ISM to modify starch is worth exploring. Clarifying the modification effect of ISM on rice starch can provide new ideas for the modification of rice starch, and broaden the application of rice starch in the development of rice-based products. Therefore, this study focused on examining the modification effects of ISM on its multi-layered structure and physicochemical properties. The structural modifications were analyzed through morphology, particle size distribution, damaged starch content, long-range crystalline structure, and short-range ordered structure. At the same time, the effects of ISM treatment on the thermal properties, pasting, and rheological properties of rice starch were studied.

## 2. Materials and Methods

### 2.1. Material

Rice starch was obtained from Jiangxi Jinong Biotechnology Co., Ltd. (Yichun, China), and it had a moisture content of 5.32% (*w*/*w*), 0.66% fat, and 0.28% protein [16]. The starch damage assay kit was purchased from Megazyme International Ireland Ltd. (Wicklow, Ireland). Potassium bromide of chromatographic grade was supplied by Aladdin Reagents Co., Ltd. (Shanghai, China). All other chemical reagents used in the study were of analytical grade and were also procured from Aladdin Reagents Co., Ltd. (Shanghai, China).

### 2.2. ISM Treatment of Rice Starch

Rice starch was dispersed in distilled water and stirred to form a 6% (*w*/*v*) suspension. The suspension was then subjected to ISM treatment at pressures of 30, 60, 90, and 120 MPa in succession, respectively. Samples were collected once the treatment pressure stabilized, ensuring that the collected samples were treated under the respective pressure. The process of ISM treatment contributed to an increase in temperature of rice starch suspensions due to acute change of pressure in a very short time. The initial temperature of the suspension was 21.5 °C, and the corresponding temperature of the collected suspensions treated by ISM pressure at 30, 60, 90, and 120 MPa were 30.6, 38.4, 42.3, and 46.9 °C, respectively. In order to eliminate the effect of temperature on starch, the starch samples were immediately cooled in an ice bath to room temperature, then frozen, lyophilized, and ground. The rice starch treated at 30, 60, 90, and 120 MPa pressures was designated as ISM-30 RS′, ISM-60 RS′, ISM-90 RS′, and ISM-120 RS′, respectively. Untreated rice starch at the same concentration was freeze-dried and labeled as NRS, which was used as the control. The obtained ISM-treated rice starch (ISM-treated RS′) and NRS samples were stored in a desiccator for further analysis.

### 2.3. Scanning Electron Microscopy (SEM)

ISM-treated starch was mounted onto one side of double-adhesive tape attached to a circular specimen stub, and then sputter-coated with a thin film of gold. The morphological characteristics of rice starch were examined using an environmental scanning electron microscope (Quanta-200, FEI, Eindhoven, The Netherlands) at an accelerating voltage of 5.0 kV and a magnification of ×5000 [17].

### 2.4. Granule Size Distributions

The particle size distributions of the ISM-treated RS′ (expressed as volume percentage) were determined using a Malvern MasterSizer 3000 particle size analyzer (Malvern Instruments Ltd., Malvern, UK) following the method described in He et al. [18]. The samples were dispersed in anhydrous ethanol and subsequently introduced into the diffraction cell. Measurements were initiated once the shading rate reached 15%. The particle size was expressed as the volume-weighted mean diameter (D_[3,4]_).

### 2.5. Determination of Damaged Starch Content

The degree of starch damage in the ISM-treated RS′ was quantified using the Megazyme starch damage assay kit, following the procedure outlined in Method 76-31 from the American Association of Cereal Chemists (AACC). The damaged starch content was calculated according to the methods described in previous studies [19,20].

### 2.6. X-Ray Diffraction (XRD) Analysis

Prior to XRD analysis, samples were stored in a desiccator where a saturated solution of NaCl maintained a constant relative humidity of 75% at 25 °C for 1 week. X-ray diffractograms of ISM-treated RS′ were obtained using a Bruker D8 Advance X-ray diffractometer (Bruker, Berlin, Germany) with Cu Kα radiation at 40 kV and 40 mA. The diffraction patterns were collected over a 2θ range of 2° to 50° with a step size of 0.02° per second. The amorphous area and crystalline area were integrated between 3° and 40° (2θ) using Origin software (Version 21.0, Chicago, IL, USA), and the relative crystallinity (RC) was calculated as the ratio of the crystalline area to the total area of the diffractograms [21,22].

### 2.7. Fourier Transform Infrared (FTIR) Spectroscopy Analysis

The short-range molecular structure of starch was analyzed using a Nicolet iS10 FT-IR Spectrometer (Thermo Scientific, Waltham, MA, USA). ISM-treated starch was placed on the surface of a multi-bounce plate of Zn-Se crystal in the attenuated total reflectance (ATR) detector for measurement. Background correction was performed using an air spectrum before each sample scan. The analysis was conducted at 25 °C with a resolution of 4 cm^−1^ and an accumulation of 32 scans. FTIR spectra were recorded in transmission mode over the range of 650–4000 cm^−1^, and data were processed using OMNIC software Version 8.2. The absorption peaks between 800 and 1200 cm^−1^ in the infrared spectrum were associated with stretching vibrations of C-O and C-C, and the characteristic peaks at 1047 cm^−1^ and 1022 cm^−1^ indicated the ordered and amorphous structures of starch, respectively [5]. Therefore, spectra were baseline-corrected, and the region between 1200 and 800 cm^−1^ was deconvoluted. The selected half-band width and enhancement factor were 40 cm^−1^ and 1.9, respectively. The absorbance ratio at 1047 cm^−1^/1022 cm^−1^ was calculated to assess the impact of ISM treatment on the short-range ordered structure of starch [23].

### 2.8. Differential Scanning Calorimetry (DSC) Analysis

The thermal properties of ISM-treated RS′ samples were evaluated using a differential scanning calorimeter (DSC, 7000X, Hitachi, Tokyo, Japan) with some modifications to the procedure described in Liu et al. [24]. Approximately 3 mg of starch (dry basis) was accurately weighed and placed into an aluminum pan. Distilled water was added to the pan using a microsyringe to obtain a starch/water mass ratio of 1:3 (*w*/*w*). The pan was then sealed and allowed to equilibrate at room temperature for 8 h. Subsequently, the sample was heated from 20 °C to 100 °C at a rate of 10 °C/min under a continuous flow of dry N_2_ gas with an empty aluminum pan as a reference. The DSC curves were analyzed using the instrument software (Thermal Analysis software Version 11.2, Hitachi Corp., Japan) to determine the onset temperature (*T_o_*), peak temperature (*T_p_*), conclusion temperature (*T_c_*), and gelatinization enthalpy (Δ*H*), which characterized the thermal properties of starch. The degree of gelatinization (DG) was calculated using the following formula:

$$\mathrm{DG}\;(\%)=\frac{\Delta H-\Delta H_{\mathrm{ISM}}}{\Delta H}\times 100$$

where Δ*H* and Δ*H*_ISM_ are the gelatinization enthalpy values for native and ISM-treated starch, respectively.

### 2.9. Rapid Visco Analyzer (RVA) Analysis

The pasting properties of starch were evaluated using a Rapid Visco Analyzer (RVA, Perten Instruments, Warriewood, Australia) by referencing the AACC-approved Method 76-21 [25]. ISM-treated RS′ (3.0 g, dry basis) was added to an RVA aluminum canister containing 25 mL of deionized water. The mixture was stirred with a paddle to ensure uniform dispersion of the starch. RVA analysis was performed using the “Standard 2” program, The sample was held at 50 °C for 1 min, followed by an increase in temperature at a rate of 6 °C/min until it reached 95 °C. The sample was maintained at 95 °C for 5 min, then cooled to 50 °C at the same rate and held for 2 min to develop the final viscosity. During the process, the paddle was rotated at 960 rpm for the first 10 s and then reduced to 160 rpm for the remaining duration. The impact of ISM treatment on the pasting properties of rice starch was assessed by analyzing parameters such as peak viscosity (PV), trough viscosity (TV), final viscosity (FV), breakdown (BD), and setback (SB).

### 2.10. Rheological Properties Analysis

① Dynamic rheological measurements

Dynamic rheological measurements of ISM-treated RS′ obtained from RVA were performed using an MCR 302 rheometer (Anton Paar, Graz, Austria). A stainless steel cone–plate geometry with a 40 mm diameter, 1° cone angle, and 0.102 mm gap was used for the measurements. An appropriate amount of starch paste was withdrawn and transferred onto the rheometer plate, and excess paste was removed with a rubber spatula after pressing down the stainless steel cone–plate geometry. The sample was allowed to rest at 25 °C for 2 min before the tests. A dynamic amplitude sweep was performed at a constant frequency of 10 rad/s to determine the linear viscoelastic region of all samples. Subsequently, a dynamic frequency sweep was conducted within the frequency range of 0.01 to 10 Hz at a constant strain of 0.5% (within the linear region) to assess the storage modulus (G′), loss modulus (G″), and loss factor (tanδ) as a function of frequency.

② Steady shear test: in-shear structural recovery measurements

Shear structural recovery of ISM-treated RS′ was measured at 25 °C using a CC27 concentric cylinder measuring system (SN 36390) by referencing the method of Liu et al. [26]. The in-shear structural recovery measurements were performed as follows: (1) the sample was sheared at a constant rate of 1 s^−1^ for 120 s; (2) a constant shear rate of 300 s^−1^ was applied for 60 s; (3) the sample was sheared at a constant rate of 1 s^−1^ for 180 s. The in-shear recovery value was calculated as the ratio of the average apparent viscosity (η) obtained during the first 120 s of the third stage to the average viscosity (η) measured in the first stage.

### 2.11. Data Analysis

All experiments were performed in triplicates, and three repetitions were conducted using three samples per experiment. The mean values and standard deviations were calculated using SPSS 25.0 statistical analysis software (Chicago, IL, USA). Significant differences between sample means were assessed using one-way analysis of variance (ANOVA), followed by Duncan’s multiple range test (*p* < 0.05).

## 3. Results and Discussion

### 3.1. Morphology

**Figure 1:**
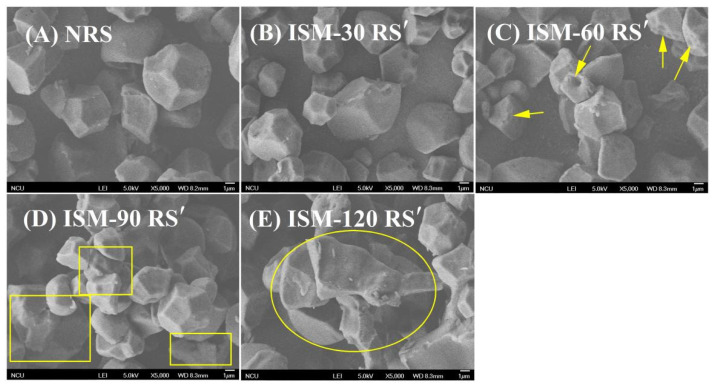
Scanning electron micrographs of NRS and ISM-treated RS′. (**A**) NRS; (**B**) ISM-30 RS′; (**C**) ISM-60 RS′; (**D**) ISM-90 RS′; (**E**) ISM-120 RS′.

The morphology changes of rice starch after ISM treatment were examined using scanning electron microscopy (SEM) (Figure 1). The results demonstrated that ISM treatment substantially altered the surface morphology of rice starch. The native starch (NRS) granules were of an integral polygonal shape (Figure 1A). No significant changes in the shape or surface characteristics of starch granules were observed after treatment at 30 MPa (Figure 1B). However, as the treatment pressure increased, notable changes in morphology became apparent. Some granules of ISM-60 RS′ exhibited surface grooves, and aggregation was observed (yellow arrows in Figure 1C). For ISM-90 RS′, a more pronounced aggregation of granules was observed (yellow rectangles, Figure 1D), while ISM-120 RS′ displayed substantial damage with some granules losing their original shape and forming aggregated block-like structures (yellow circles, Figure 1E). The morphology disruption induced by ISM treatment was similar to that reported in previous studies [27]. The higher the pressure applied during ISM treatment, the stronger the mechanical forces and effects, which led to an increased degree of starch destruction. Previous studies have indicated that the forces generated by ISM caused pea starch granules to swell without fracturing [11], while larger potato starch granules first swelled and then fractured under ISM treatment [7]. In the present study, ISM treatment resulted in the aggregation of smaller rice starch particles, suggesting that ISM treatment induced varying degrees of damage to starch granules with different sizes. This could be attributed to the differential collision, impact, and frictional forces experienced by starch granules of various sizes during the ISM treatment process.

### 3.2. Granule Size Distributions

**Figure 2:**
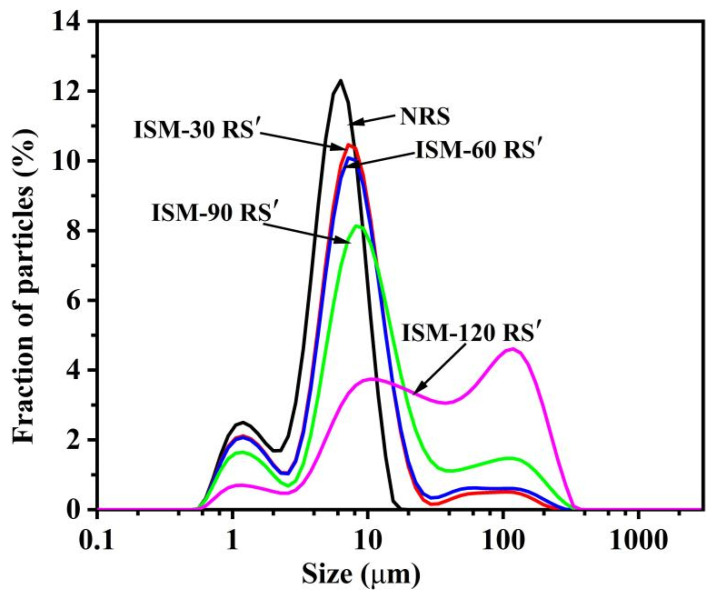
Particle size distribution of NRS and ISM-treated RS′.

As discussed in Section 3.1, where rice starch aggregation was observed after ISM treatment, it was found that ISM treatment resulted in a shift in granule size distribution peak toward larger particle sizes. As illustrated in Figure 2, the granule size distribution curve of the untreated rice starch (NRS) exhibited a bimodal pattern, with a small peak around 1 μm. This observation aligned with the distribution patterns previously reported for rice starch from different genotypes [28], but differed from that of rice starch with a monomodal distribution reported by Li et al. [29]. It was likely related to the starch granule characteristic induced by extraction. After ISM treatment, a notable change in the granule size distribution curve was observed. As the treatment pressure increased, the curve progressively shifted to the right with a prominent peak around 100 μm, resulting in a trimodal distribution. Concurrently, the peak around 1 μm diminished, while the peak at approximately 6.51 μm broadened and shifted to the right. These findings suggested that ISM treatment led to an increase in the granule size of rice starch. The D_[4,3]_ values for NRS, ISM-30 RS′, ISM-60 RS′, ISM-90 RS′, and ISM-120 RS′ were 5.9 μm, 11.8 μm, 13.7 μm, 25.0 μm, and 59.9 μm, respectively. The increase in granule size further supported the aggregation phenomenon identified in morphological observation (Figure 1). In addition to the alterations in starch morphology and granule size caused by ISM treatment, it was highly probable that the internal structure of starch was also modified.

### 3.3. Damaged Starch

After ISM treatment, starch granules underwent deformation due to shear, impact, and other forces generated by collisions between the medium and the particles, leading to alterations in their morphology, crystalline structure, and molecular configuration [30]. The starch damage content reported for rice starch by [31] (6.4%) was higher than the damage content (4.24%) observed in NRS used in this study. This discrepancy may be attributed to differences in extraction methods or variations in cultivars. As presented in Table 1, ISM treatment significantly increased the damaged starch content in rice starch. Specifically, the damaged starch content in ISM-30 RS′, ISM-60 RS′, ISM-90 RS′, and ISM-120 RS′ was 4.52%, 4.82%, 5.68%, and 17.99%, respectively. When the applied pressure was below 90 MPa, the increase in damaged starch content was relatively modest. However, ISM treatment at 120 MPa caused the damaged starch content to increase by a factor of 4.2. The trend in damaged starch content with increasing pressure mirrored that observed for potato starch [7], while the degree of damage in pea starch was comparatively lower [11]. The extent of starch damage induced by ISM treatment may be influenced by factors such as the granule size of starch and the arrangement of its crystalline structure.

### 3.4. Long-Range Crystalline Structure

**Figure 3:**
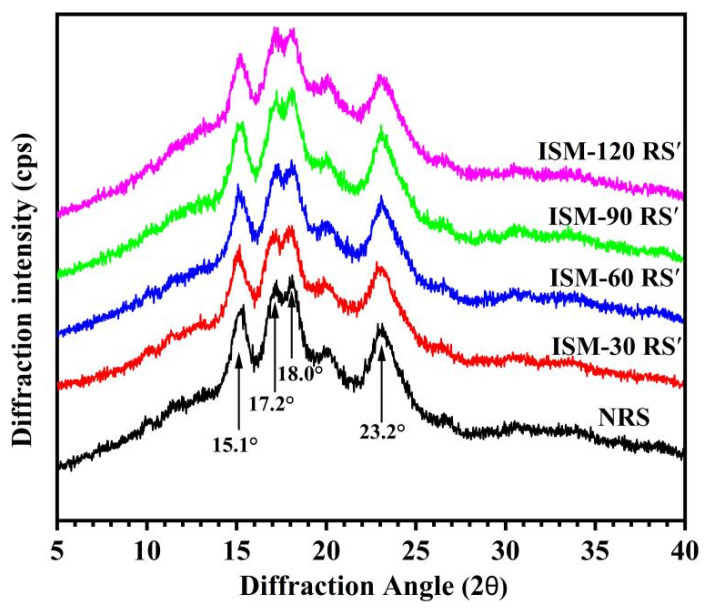
X-ray diffraction patterns of NRS and ISM-treated RS′.

Crystallinity is a fundamental aspect of starch structure, encompassing both the type of crystal and its relative crystallinity (RC). According to X-ray diffraction (XRD) patterns, native starch can be classified into three crystal types: A, B, and C, where the crystal type is determined by the structure of the amylopectin side-chain double helix or the amylose single helix [32]. To examine the effect of ISM treatment on the long-range crystalline structure of rice starch, XRD analysis was performed. As shown in Figure 3, XRD patterns of ISM-treated RS′ were similar to those of native rice starch (NRS), displaying diffraction peaks at 15.1°, 17.2°, 18.0°, and 23.2° (2θ), which indicated that ISM treatment preserved the A-type crystalline structure of rice starch. However, ISM treatment resulted in a reduction in the intensity of these diffraction peaks and a decrease in the relative crystallinity of the rice starch. The RC of NRS was 29.01%, and the RC values for ISM-90 RS′ and ISM-120 RS′ were reduced to 26.46% and 20.74%, respectively. The maximum reduction in crystallinity was higher than that of rice starch treated with a dynamic high-pressure microfluidizer in the study by Li et al. [27]. Compared to the effect of high-pressure homogenization on the crystalline structure of maize starch, the decrease in crystallinity was also more significant [33]. These findings suggested that ISM treatment disrupted the long-range crystalline structure of rice starch, with a more pronounced disruption occurring at higher treatment pressures.

### 3.5. Short-Range Ordered Structure

**Figure 4:**
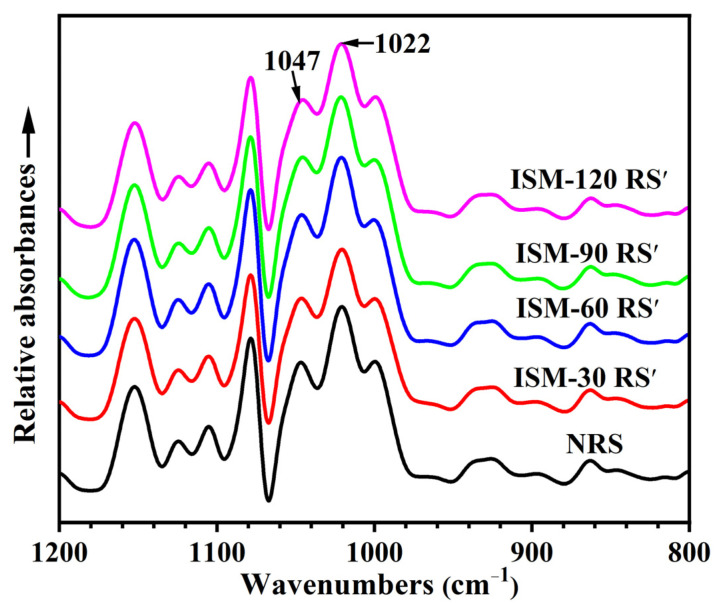
Deconvoluted FTIR spectra from 1200 cm^−1^ to 800 cm^−1^ wavenumbers of NRS and ISM-treated RS′.

The deconvoluted FTIR spectra at 1047 cm^−1^ and 1022 cm^−1^ provided insight into the short-range molecular structure of starch, reflecting the structural order of starch chains near the surface of starch granules (approximately 2 μm) [34]. Figure 4 shows the deconvoluted FTIR spectra of ISM-treated RS′ within the 1200~800 cm^−1^ range. The peak intensity at 1047 cm^−1^ for ISM-treated RS′ was diminished, and the ratio of 1047 cm^−1^/1022 cm^−1^ decreased as the treatment pressure increased. These changes suggested that ISM treatment disrupted the short-range ordered structure of rice starch. As indicated in Table 1, the 1047 cm^−1^/1022 cm^−1^ values for NRS, ISM-30 RS′, ISM-60 RS′, ISM-90 RS′, and ISM-120 RS′ were 0.88, 0.85, 0.85, 0.76, and 0.73, respectively. The reduction in the ratio of 1047 cm^−1^/1022 cm^−1^ observed in the FTIR spectra of ISM-treated RS′ may be attributed to the dissociation and disruption of the double helix structure within the crystalline regions of the starch [35]. The disruption of both the long-range and short-range structures further confirmed that ISM treatment affected the internal organization of rice starch.

### 3.6. Thermal Properties

Table 2 presented the gelatinization transition temperatures (*T_o_*, *T_p_*, and *T_c_*) and gelatinization enthalpy (Δ*H*) of ISM-treated RS. ISM treatment significantly influenced the gelatinization transition temperatures of rice starch. Compared to NRS, *T_o_*, *T_p_*, and *T_c_* values of ISM-treated RS initially decreased and then increased. Except for ISM-120 RS′, *T_o_* and *T_p_* values for ISM-30 RS′, ISM-60 RS′, and ISM-90 RS′ were lower than those of NRS. At lower treatment pressures, ISM treatment disrupted the crystalline structure of rice starch, resulting in a decrease in gelatinization temperatures. However, higher ISM treatment pressures led to an increase in gelatinization temperatures, likely due to the formation of a more perfectly packed crystalline structure in ISM-120 RS′, thereby resulting in higher transition temperatures [17]. As treatment pressure increased, Δ*H* of ISM-treated RS′ decreased. Δ*H* values for NRS, ISM-30 RS′, ISM-60 RS′, ISM-90 RS′, and ISM-120 RS′ were 6.61, 6.55, 6.52, 5.90, and 4.53 mJ/mg, respectively. The Δ*H* value was proportional to the degree of double-helix structure in the internal regions of the starch, and a greater mechanical disruption of the double-helix structure resulted in lower enthalpy [36]. Furthermore, damage to the crystalline regions of starch led to a reduction in gelatinization enthalpy [29]. Based on the gelatinization enthalpy, the degree of gelatinization (DG) for ISM-treated RS′ was calculated. It was shown that the gelatinization degree of ISM-treated RS′ increased with rising treatment pressure, with DG reaching 31.39% for ISM-120 RS′. The DSC results confirmed that starch gelatinization involved the disruption of granules, the breakdown of crystalline structures, and the dissociation of helical structures. The applied pressure played a crucial role in ISM-induced starch gelatinization process.

### 3.7. Pasting Properties

**Figure 5:**
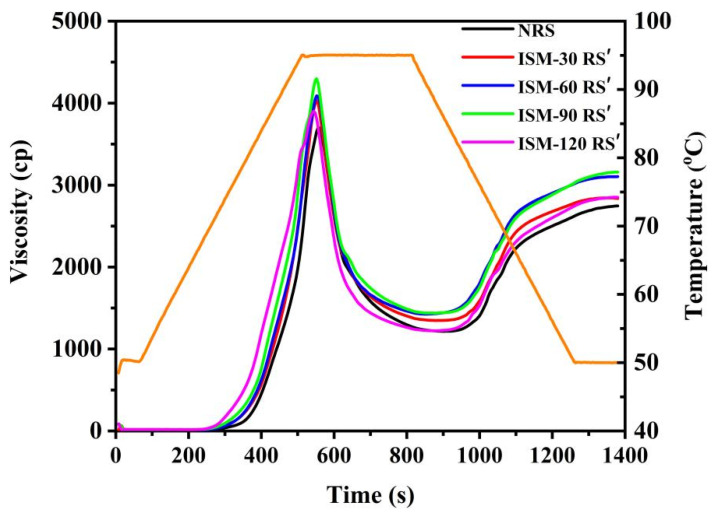
Pasting properties of NRS and ISM-treated RS′. The orange line represents the change of temperature with time during RVA test.

Starch gelatinization is influenced by various factors, including amylose leaching, granule swelling, friction between swollen granules, the crystallinity of starch components, and chain length [37]. Figure 5 illustrates the gelatinization curves of ISM-treated RS′, while Table 3 presents the corresponding gelatinization parameters. As the temperature increased, starch granules in the RVA underwent stirring and friction between the granules, which caused them to absorb water and swell. The leaching of amylose further elevated viscosity, which subsequently decreased at higher temperatures, leading to a trough viscosity (TV) due to accelerated thermal motion. Upon cooling, amylose molecules re-associated, resulting in short-term retrogradation and a viscosity increase at 50 °C, which formed the final viscosity (FV) [38]. Compared to NRS, peak viscosity (PV), trough viscosity (TV), and final viscosity (FV) values of ISM-treated RS′ all increased (Table 3), indicating that ISM treatment enhanced the swelling capacity, resistance to disruption, and gelling ability of rice starch. Consequently, breakdown (BD) and setback (SB) values for ISM-treated RS′ also increased, suggesting that ISM treatment promoted short-term retrogradation of rice starch. It was conducive to rapidly form gel structure for maintain shape in the production process of rice noodles. In contrast to the findings of this study, a previous report indicated that conventional dynamic high-pressure microfluidization treatment reduced the pasting viscosity of rice starch [27], implying that the effects of industry-scale microfluidizer differed from those of conventional dynamic high-pressure microfluidization modification of starch. As the treatment pressure increased, the pasting viscosity of rice starch initially increased and then decreased, while the highest viscosity was observed in ISM-90 RS′. For example, peak viscosity (PV) values for NRS, ISM-30 RS′, ISM-60 RS′, ISM-90 RS′, and ISM-120 RS′ were 3740.7, 4072.7, 4053.0, 4317.7, and 3914.0 cp, respectively. The observed viscosity changes during pasting were likely associated with the extent of starch granule damage. Treatment at 120 MPa resulted in the majority of starch granules fracturing and aggregating into clusters (Figure 1E). The fractured starch granules exhibited a reduced capacity for water absorption and swelling during gelatinization, leading to a decrease in pasting viscosity.

### 3.8. Rheological Properties

#### 3.8.1. Dynamic Viscoelastic Rheological Properties

**Figure 6:**
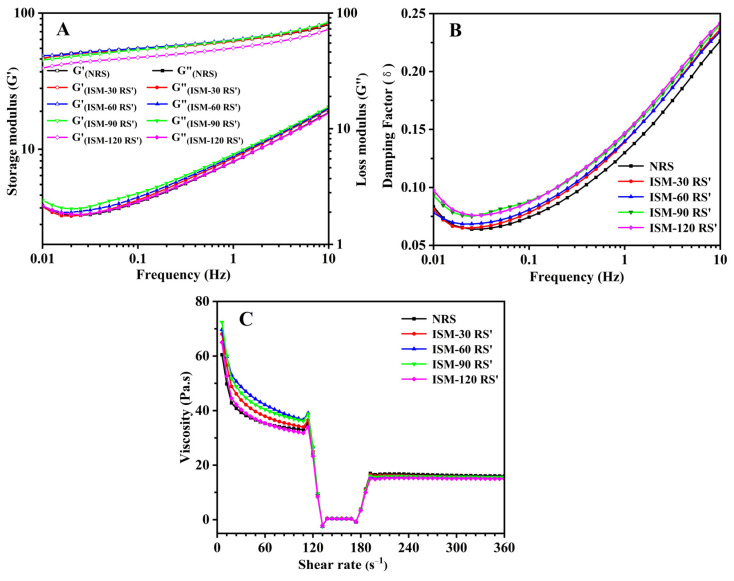
Dynamic viscoelastic moduli (**A**), loss factor (**B**), and in-shear recovery curves (**C**) of NRS and ISM-treated RS′.

Figure 6 illustrates the changes in the storage modulus (G′), loss modulus (G″), and loss factor (tanδ) of ISM-treated RS′ over a frequency range of 0.1 to 10 Hz. As displayed in Figure 6A, both G′ and G″ exhibited frequency dependence, and values of modulus increased as the frequency increased. Additionally, G′ was significantly higher than G″, indicating that NRS and ISM-treated RS′ gels primarily exhibited elastic rather than viscous behavior. After ISM treatment, the G’ value of rice starch experienced a slight decrease. The dynamic viscoelastic rheological properties of starch gels were influenced by several factors, including the crosslinking density of the continuous phase, rigidity, entanglement between amylose and amylopectin, spatial distribution, and the effective interactions within the dispersed phase [39]. Although ISM treatment disrupted both the long-range crystalline structure and short-range ordered structure of starch, it did not completely break the starch molecules or significantly weaken the crosslinked gel network, thus preventing a substantial decrease in the elastic modulus. Furthermore, ISM treatment did not significantly enhance the G″ value of rice starch. No significant trends were observed in the changes of G′ and G″ as treatment pressure increased.

As shown in Figure 6B, the tanδ values for both NRS and ISM-treated RS′ were less than 1, indicating that both gels exhibited predominantly elastic behavior. Tanδ is commonly used to assess changes in viscoelastic properties related to interactions between components. For example, the destruction of the crosslinked network typically results in a rapid increase in tanδ [40]. ISM treatment slightly increased the tanδ value of rice starch, suggesting that ISM treatment mildly weakened the crosslinked network structure of rice starch gels, which may be related to slight molecular degradation, as explained by Wang et al. [41].

#### 3.8.2. In-Shear Structural Recovery Properties

The in-shear structural recovery measurement was employed to assess the ability of rice starch gels to restore their original structure under low-shear conditions after being subjected to high shear stress [42]. Figure 6C shows the shear recovery curves for NRS and ISM-treated RS′ gels, and Table 3 summarizes the shear recovery values. Under high shear conditions (300 s^−1^), the viscosity of all starch gels significantly decreased, indicating disruption of the gel structure. During the subsequent low shear recovery phase (1 s^−1^) over 120 s, no significant differences in shear viscosity were observed among the samples. The shear recovery values for NRS, ISM-30 RS′, ISM-60 RS′, ISM-90 RS′, and ISM-120 RS′ were 42.9%, 41.0%, 38.5%, 38.1%, and 40.7%, respectively, suggesting that ISM treatment did not notably affect the shear recovery capacity of rice starch gels. As discussed by [38], shear recovery was influenced by factors such as the dissolution of amylopectin molecules and the degree of crosslinking between starch molecules. As highlighted in Section 3.8.1 Dynamic Viscoelastic Rheological Properties on dynamic viscoelastic rheology, ISM treatment likely did not induce starch molecule breakage or significantly weaken the crosslinking between starch molecules, thus not altering the shear recovery properties of rice starch gels.

## 4. Conclusions

The study demonstrated that ISM treatment significantly modified the granule morphology, crystalline structure, and short-range ordered structure of rice starch. Specifically, starch granules aggregated, while the long-range crystallinity structure and short-range ordered structure were weakened. Additionally, the gelatinization enthalpy of rice starch decreased, and the degree of gelatinization increased. The gelatinization of rice starch induced by ISM treatment served as the primary factor underlying the observed changes in starch granule morphology, long-range crystallinity, and short-range ordered structure. Although ISM treatment disrupted the internal structure of starch, leading to an increase in RVA pasting viscosity, it did not significantly affect the dynamic rheological moduli, loss factor, and shear recovery values. These findings suggested that ISM modification had the potential to produce rice starch with a disordered structure, enhanced viscosity, and a preserved gel network, which may be advantageous for the application of industry-scale microfluidization in the development of products such as pie fillings, baby foods, and salad dressings that require thickening and resistance to shear.

## Figures and Tables

**Table 1:** Structural properties of NRS and ISM-treated RS′ ^a^.

Samples	D_[4,3]_	Damaged Starch Content (%)	Relative Crystallinity (%)	1047 cm^−1^/1022 cm^−1^
NRS	5.9 ± 0.3 d	4.25 ± 0.07 d	29.01 ± 0.18 a	0.88 ± 0.01 a
ISM-30 RS′	11.8 ± 0.2 cd	4.52 ± 0.05 cd	28.33 ± 0.18 a	0.85 ± 0.03 b
ISM-60 RS′	13.7 ± 0.5 c	4.82 ± 0.08 c	27.78 ± 0.11 a	0.85 ± 0.02 b
ISM-90 RS′	25.0 ± 0.6 b	5.68 ± 0.06 b	26.46 ± 0.45 b	0.76 ± 0.02 c
ISM-120 RS′	59.9 ± 3.1 a	17.99 ± 0.40 a	20.74 ± 0.47 c	0.73 ± 0.01 d

^a^ Different letters in the same column indicate significant differences between samples (*p* < 0.05).

**Table 2:** Thermal properties of NRS and ISM-treated RS′ ^a^.

Samples	*T_o_* (°C)	*T_p_* (°C)	*T_c_* (°C)	Δ*H* (mJ/mg)	DG (%)
NRS	61.67 ± 0.07 b	66.40 ± 0.27 a	73.28 ± 0.07 a	6.61 ± 0.04 a	0
ISM-30 RS′	60.07 ± 0.15 c	63.74 ± 0.14 b	71.60 ± 0.11 a	6.40 ± 0.19 ab	3.10 ± 2.88 c
ISM-60 RS′	59.91 ± 0.08 c	63.84 ± 0.00 b	70.64 ± 0.30 a	6.22 ± 0.13 bc	5.90 ± 1.92 bc
ISM-90 RS′	60.06 ± 0.43 c	64.13 ± 0.85 b	70.69 ± 2.40 a	5.90 ± 0.23 c	10.74 ± 3.42 b
ISM-120 RS′	63.21 ± 0.01 a	66.96 ± 0.02 a	72.34 ± 0.16 a	4.53 ± 0.06 d	31.39 ± 0.96 a

^a^ Different letters in the same column indicate significant differences between samples (*p* < 0.05).

**Table 3:** Pasting parameters of NRS and ISM-treated RS′ ^a^.

Samples	PV (cp)	TV (cp)	BD (cp)	FV (cp)	SB (cp)	In-Shear Structural Recovery (%)
NRS	3740.7 ± 35.7 d	1231.0 ± 13.5 d	2509.7 ± 22.4 d	2756.7 ± 36.2 c	1525.7 ± 38.7 c	42.9 ± 1.1 a
ISM-30 RS′	4072.7 ± 40.5 b	1361.3 ± 12.7 c	2711.3 ± 32.0 b	2890.3 ± 45.8 b	1519.0 ± 26.6 c	41.0 ± 2.8 a
ISM-60 RS′	4053.0 ± 46.7 b	1411.7 ± 23.1 b	2641.3 ± 24.7 c	3083.7 ± 45.1 a	1672.0 ± 22.1 ab	38.5 ± 5.4 a
ISM-90 RS′	4317.7 ± 37.6 a	1445.7 ± 16.9 a	2872.0 ± 20.8 a	3136.3 ± 19.6 a	1690.7 ± 31.0 a	38.1 ± 2.6 a
ISM-120 RS′	3914.0 ± 15.6 c	1222.0 ± 0.0 d	2692.0 ± 15.6 b	2848.5 ± 3.5 b	1626.5 ± 3.5 b	40.7 ± 1.3 a

^a^ Different letters in the same column indicate significant differences between samples (*p* < 0.05).

## Data Availability

The original contributions presented in this study are included in the article. Further inquiries can be directed to the corresponding author.
